# Clinical and neuroimaging correlates of cognition in HIV

**DOI:** 10.1007/s13365-019-00763-w

**Published:** 2019-06-18

**Authors:** Olubanke Davies, Becky I. Haynes, Sarah J. Casey, Sofia Gerbase, Gareth J. Barker, Mervi Pitkanen, Ranjababu Kulasegaram, Michael D. Kopelman

**Affiliations:** 1 Department of Genitourinary & HIV Medicine, Guy’s and St Thomas’ Hospitals NHS Trust, Great Maze Pond, London, SE1 9RT UK; 2 King’s College London (Institute of Psychiatry, Psychology & Neuroscience), London, UK; 3 South London and Maudsley NHS Foundation Trust based at St Thomas’ Hospital, London, UK; 4 Biomedical Research Centre of the Institute of Psychiatry, Psychology, and Neuroscience, London, UK

**Keywords:** HIV, Cognition, Anxiety, Aging, MRI, DTI

## Abstract

This study investigated whether HIV-positive participants, stable on combined antiretroviral therapy (cART), showed cognitive impairments relative to HIV-negative controls; and whether clinical and neuroimaging factors correlated with cognitive function in the HIV-positive participants. One hundred and twenty-six white men who have sex with men, of whom 78 were HIV-positive and stable on cART and 48 were HIV negative, were recruited to this cross-sectional study. The median age of HIV-positive participants in this study was 47. They underwent clinical and neuropsychological evaluation and magnetic resonance imaging of the brain, including diffusion tensor imaging (DTI). Cognitive scores for both groups were compared, and regression models were run to explore the influence of clinical, psychiatric, lifestyle, and neuroimaging variables on cognition. The prevalence of neurocognitive impairment, using the multivariate normative comparison criteria, was 28% in HIV-positive participants and 5% in HIV-negative participants. After covarying for age, years of education, and non-English speaking background, there were significant differences between the HIV group and the controls across four cognitive domains. The HIV group showed significantly higher mean diffusivity (MD) and lower fractional anisotropy (FA) than the control group on DTI. Although anxiety levels were clinically low, anxiety and DTI measures were the only variables to show significant correlations with cognitive function. In the HIV group, poorer cognitive performance was associated with higher MD and lower FA on DTI and higher (albeit clinically mild) levels of anxiety. Our findings suggest that white matter changes and subtle anxiety levels contribute independently to cognitive impairment in HIV.

## Introduction

The introduction of combined antiretroviral therapy (cART) has led to a dramatic decline in HIV-associated mortality and morbidity. However, a significant proportion of patients continue to experience cognitive deficits with reported prevalence rates of 20–50% depending on the precise selected cut-offs (Heaton et al. 2010; Valcour et al. 2004; Robertson et al. 2004; Elbirt et al. 2012; Ku et al. 2014; Marin-Webb et al. 2016; Underwood et al. 2017; Parry et al. 2017). Since the advent of cART, these cognitive deficits have taken a milder form (Heaton et al. 2010; Antinori 2007; Pfefferbaum et al. 2014; Janssen et al. 2015, 2017; Cole et al. 2018), but continue to impact negatively on quality of life (Heaton et al. 2004) with marked effects on patients and their families and significant cost implications for health and social services.

Although some studies have found no significant differences between HIV-positive and HIV-negative control participants on neuropsychological tests (Dawes et al. 2008; Towgood et al. 2012), the persistence of HIV-associated neurocognitive disorder (HAND) in other studies (Becker et al. 2009) may be because people are living longer with HIV. Moreover, the central nervous system (CNS) is recognized as a quiescent reservoir of the virus, and antiretroviral (ARV) entry into the brain is variable because of the blood-brain barrier. Cognitive decline in HIV may, therefore, relate to the insufficient CNS penetrance of some antiretroviral drugs (Robertson et al. 2004; Fiandra et al. 2017). Antiretroviral drugs have been classified according to their cerebral penetration effectiveness (Letendre et al. 2010), but there are conflicting reports on their protective effect on cognition (Robertson et al. 2004; Antinori et al. 2004). It has also been proposed that a cART-insensitive self-sustaining immunological response to the infection may contribute to HIV-associated neurocognitive impairment (Avison et al. 2004; Pu et al. 2007; Cardenas et al. 2009; Harezlak et al. 2011; Hong and Banks 2015).

Risk factors associated with HIV-associated cognitive impairment include longer duration of HIV (Robertson et al. 2007), older age (Valcour et al. 2006; Paddick et al. 2017), low nadir CD4 count (Heaton et al. 2010), CD4 count less than 350 (Bhaskaran et al. 2008), persistent viremia (Heaton et al. 2010), substance abuse (Nath 2010; Patel et al. 2013), lower educational level (Paddick et al. 2017; De Ronchi et al. 2002), and hepatitis C coinfection (Parsons et al. 2006; Sun et al. 2013).

The presence of cognitive impairment affects everyday functioning (Heaton et al. 2004) and has detectable neuroimaging correlates, particularly in the frontal and parietal lobes (Thompson et al. 2005; Thompson et al. 2006). Moreover, Chang et al. (2008) found that HIV-positive individuals showed a greater increase in mean diffusivity (MD) in the genu of the corpus callosum compared with an HIV-negative control group. Our own previous study (Towgood et al. 2012), comparing cognitively asymptomatic HIV-positive with matched HIV-negative participants, did not reveal any significant differences on neuropsychological tests after Bonferroni correction. However, there was reduced frontal gray matter volume on magnetic resonance imaging (MRI) in the HIV-positive participants (Towgood et al. 2012) and evidence of anterior cingulate change on arterial spin labeling and positron emission tomography (Towgood et al. 2013). Other investigations have described reduced gray and white matter in HIV-positive participants (Thompson et al. 2005; Becker et al. 2012; Kuper et al. 2011; Chiang et al. 2007). Underwood et al. (2017) found lower total gray, but not white, matter volume in HIV-positive participants than in controls, as well as lower fractional anisotropy (FA) and higher mean and radial diffusivity on diffusion tensor imaging (DTI). In that study, reduced gray matter volume and lower FA were associated with poorer cognition. Li et al. (2018) compared DTI in cART-untreated patients and healthy volunteers, finding pronounced changes in the corpus callosum and corona radiata. Alterations in the corona radiata correlated with CD4/CD8 ratio. Increased MD was significantly associated with impairments in many cognitive domains. Another study (Janssen et al. 2015) found lower brain parenchymal fraction and thalamic volume in HIV-positive participants, with reduced speed of information processing and verbal fluency at one-year follow-up (Janssen et al. 2017). In our own longitudinal study (Haynes et al. 2018), we found significant effects of both HIV and age on longitudinal changes in cognitive and MD measures, but not on changes in the gray and white matter volumes. Although many studies have investigated white matter integrity in HIV and the contribution of clinical variables, findings show quantitative inconsistencies (O’Connor et al. 2017).

The aim of the present study was to investigate the prevalence of cognitive impairment in HIV-positive participants and to examine its quantitative association with clinical and MRI measures. The HIV-positive individuals were on established antiretroviral therapy, with undetectable HIV viral loads.

We hypothesized that:

(i) There would be a difference in mean cognitive scores and prevalence of cognitive impairment between the HIV-positive participants and their controls.
(ii) There would be MRI and DTI differences between the two groups.
(iii) Metabolic comorbidities, HIV severity and duration, concomitant alcohol or substance use, psychiatric state (anxiety, depression), and MR findings would each contribute to cognitive impairments in HIV-positive participants.

## Methods

We employed a cross-sectional study utilizing a between-groups design, where the primary independent variable was HIV status. The primary dependent variable was cognitive performance on neuropsychological tests. Other variables included mood state, length of time diagnosed with HIV, metabolic comorbidities, length of time on cART, length of time without cART following diagnosis, CNS penetration effectiveness (CPE) score of their cART, nadir and current CD4 count, and diffusion tensor brain imaging metrics. Treatment duration and length of time without cART following diagnosis was based on patient report, confirmed (in most cases) from the medical records. CPE score was assigned using a published ranking system (Letendre et al. 2008, 2009) and was utilized as a continuous variable. Participants were recorded as having a “metabolic comorbidity” if they had hypertension, hyperlipidemia, or diabetes mellitus requiring pharmacological treatment.

We recruited to the HIV group 78 white males proficient in the English language (see Table 1) and aged 25 to 74, who self-reported as “men who have sex with men.” They were stable on cART (i.e., no change in the combination of medications or disease markers) for > 6 months, with HIV viral load < 50 copies/ml. The control group consisted of 48 white males proficient in the English language and aged 26 to 76 who also self-reported as “men who have sex with men,” but were HIV negative. The study groups overlapped with the Haynes et al. participants (Haynes et al. 2018), but also included further HIV cases and controls, aged 25–76. Unlike the Haynes et al. study (2018), we included patients and controls aged between 40 and 50, such that there was a continuous range of ages in this sample. As a result, the median age in this sample of HIV patients was 47, but this allowed us to look at the effect of HIV on aging across a range of ages. To allow greater variability in the sample, we also included participants with intermittent (non-dependent) alcohol or substance use or with mild cognitive complaints.

**Table 1:** Demographic and clinical variables

	Control (*n* = 48)	HIV (*n* = 78)	*p*
Age	43.73 (13.51)	46.87 (12.38)	0.184
Years in education	16.42 (1.76)	14.97 (2.46)	< 0.001
NESB	10 (21%)	8 (10%)	0.099
FSIQ	121.42 (11.57)	113.19 (13.42)	0.001
BDI	4.90 (3.39)	7.99 (7.36)	0.002
BAI	3.67 (3.26)	6.33 (7.16)	0.005
PRMQ–prospective	56.81 (9.14)	51.27 (10.84)	0.004
PRMQ–retrospective	58.62 (7.75)	54.53 (9.37)	0.013
Alcohol units per week	11.59 (9.18)	11.48 (9.29)	0.948
Drug use in past 3 months	8 (17%)	18 (23%)	0.388
CD4 current		691.38 (280.58)	
CD4 nadir		210.09 (128.64)	
Illness duration^1^		11.22 (6.52) (range 1–31)	
Treatment duration^1^		8.27 (5.05)	
Time without treatment^1^		2.95 (3.93)	
CPE score		6.73 (1.48)	
Number diagnosed pre cART (< 1995)		11 (14%)	
Number with metabolic comorbidities		18 (23%)	

*NESB*, non-English speaking background; *FSIQ*, full scale IQ calculated using the Weschler Adult Intelligence Scale-III short form (Crawford 2008); *BDI*, =Beck Depression Inventory; *BAI*, =Beck Anxiety Inventory; *PRMQ*, Prospective and Retrospective Memory Questionnaire; *CPE*, CNS penetration effectiveness

Metabolic comorbidities: diabetes, anti-hypertensive medication, hypercholesterolemia medication

^1^Time in years

Exclusion criteria were: clinical AIDS (AIDS defining illness) whether or not the CNS was involved; confounding neurological disorder diagnoses (e.g., multiple sclerosis, stroke, Alzheimer’s disease, other degenerative brain diseases, any brain infection, or neoplasm); major head trauma; significant alcohol or recreational drug use (> 25 units of alcohol per week; more than weekly cannabis use, and more than monthly use of cocaine and amphetamines); chronic medical illness that might affect cognition (e.g., chronic hepatitis C, chronic hepatitis B, uncontrolled hypertension, abnormal thyroid function, or diabetes); severe cardiac, hepatic, or renal dysfunction; or history of serious psychosis or major depression. One HIV patient was excluded because he was diagnosed with a space-occupying lesion shortly after the assessment. Another patient was excluded from the MRI analyses because of a venous malformation but, as this was clinically asymptomatic, he was included in the cognitive analyses. These exclusion criteria meant that the cohort might represent an unusually healthy group and may thus not be a representative of the general clinic population. These criteria were selected to minimize the number of confounding factors.

Informed consent was obtained from all participants according to the Declaration of Helsinki and the study was approved by the East London Research Ethics Committee (Ref. 11/LO/0037).

The following variables were measured:

### Clinical evaluation

All participants underwent routine blood investigations including HIV viral load, CD4/CD8 count, syphilis, hepatitis B and C, and glucose levels. The Beck Depression Inventory II (BDI-II) (Beck 1987) and Beck Anxiety Inventory (BAI) (Beck and Steer 1990) were used to assess mood state. The frequency of perceived memory difficulties was evaluated using the Prospective and Retrospective Memory Questionnaire (PRMQ) (Smith et al. 2000).

### Cognitive assessment

Participants were investigated with a detailed neuropsychological evaluation, administered by trained psychologists (SC, RH, SG) using standardized procedures. The cognitive test battery was broadly based on tasks used by Heaton et al. (1995). The cognitive tasks were analyzed in terms of four cognitive domains which conformed to those of the Diagnostic and Statistical Manual of Mental Disorders-5th edition (Sachdev et al. 2014).

- Executive function: Weschler Adult Intelligence Scale (third edition) Matrix reasoning and Letter number sequencing (WAIS III) (Weschler 1997); Phonemic fluency; Category fluency; Trail making test Part B minus Part A (Reitan and Wolfson 1988); Hayling test (Burgess and Shallice 1997); Brixton spatial anticipation test (Burgess and Shallice 1997); Modified card sorting test (Nelson 1976)
- Complex attention: WAIS III Digit span; WAIS III Digit symbol coding; WAIS III Symbol search (Weschler 1997); Trail making test–Part A (Reitan and Wolfson 1988); Paced Auditory Serial Addition Test (Gronwall and Sampson 1974)
- Memory: Weschler Memory Scale-Revised (Wechsler 1987), Logical memory I and II*;* Visual reproduction I and II*;* Rey Auditory Verbal Learning Test (Rey 1964)*;* Warrington Recognition Memory Test (words and faces)*;* a word-pair and single-word recognition test (Castel and Craik 2003).
- Perceptuo-motor function: WAIS III Block design and Grooved pegboard (Lafayette Instruments 2002)

In addition, the revised National Adult Reading Test ((NART-R) Nelson and Wilson 1991) was used as an estimate of premorbid IQ.

### MRI protocol

MRI of the brain was performed on a GE SIGNA 3T MR scanner (General Electric Healthcare, Chicago, USA) at the Centre for Neuroimaging Sciences, King’s College London. For volumetric analysis, a 3D volumetric scan was acquired in a sagittal orientation with parameters based on the ADNI 2 protocol (http://adni.loni.usc.edu/methods/documents/mri-protocols/) giving 1 × 1 × 1.2 mm voxels over the whole brain in approx. 6 min (TE = 2.8 ms, TR = 7.0 ms, TI = 650 ms; excitation flip angle 8 degrees; ASSET (parallel imaging speed up) factor = 2). Datasets were pre-processed and analyzed using SPM12 (Wellcome Department of Cognitive Neurology, London, UK, http://www.fil.ion.ucl.ac.uk/spm/). Individual images were segmented, and were aligned and normalized to MNI space using the DARTEL toolbox (Ashburner 2007). This analysis followed a standard protocol (Ashburner 2010) where a smoothing kernel of 10-mm full width at half maximum was applied. The groups were compared using analysis of covariance controlling for age and total intercranial volume.

DTI data were acquired using a multi-slice peripherally gated echo-planar imaging (EPI) sequence. Using an EPI pulse sequence, each DTI volume was acquired from 60 contiguous 2.4-mm-thick slices with field of view (FOV) 307 × 307 mm and matrix size 128 × 128, giving a final isotropic voxel size of 2.4 × 2.4 × 2.4 mm. Echo time was 107 ms, and effective repetition time was 12–20 R-R intervals. At each location, 4 images were acquired without diffusion weighting, together with 32 images with a weighting of 1300 s mm^−2^ applied along directions uniformly distributed in space. As acquisition of DTI data was cardiac-gated, scanning time varied according to each subject’s pulse rate, but for most subjects scanning time was between 9 and 11 min. The DTI datasets were pre-processed and analyzed using tools from the Oxford Centre for Functional MRI of the Brain Software Library. The raw DTI volumes were manually inspected to eliminate scans with large distortion or artifacts. Pre-processing used a multistep procedure that involved correction for motion and eddy currents (Jenkinson and Smith 2001), extraction of non-brain tissue and skull using a brain extraction tool (Smith 2002) and the creation of fractional anisotropy (FA) maps. No correction was made for the effect of partial volume; however, as there were no significant group differences in volume, we do not expect that the DTI results are confounded by partial volume having a larger effect in the HIV group than the controls. Maps of MD were created by averaging the apparent diffusion coefficients (ADCs) in the directions of the three principal eigenvectors.

Voxelwise analysis was performed using tract-based spatial statistics (Smith et al. 2006), part of FSL (Smith et al. 2004). Each participant’s FA map was aligned to standard space, using a nonlinear transformation, and averaged to form a mean FA image which was then thinned to form a skeleton at FA > 0.2. The aligned FA maps were projected on to the skeleton, and the HIV participants were compared with the control group using permutation-based non-parametric cluster inference (Randomize, implemented in FSL) with age added as a covariate. Results were corrected for multiple comparisons using threshold-free cluster enhancement (Smith and Nichols 2009).

### Statistical analysis

Participants’ scores on each of the cognitive measures were transformed into z-scores to permit comparison across the different domains of cognitive and neuropsychiatric functioning, using control group means and standard deviations. The scores on individual tasks were averaged to form domain scores, which were then further averaged to generate a global cognition score.

The cognitive scores for the HIV and seronegative control groups were compared using analysis of covariance, with age, years of education, and whether participants had a non-English speaking background (NESB–coded yes/no) added as covariates. Statistical analysis was carried out using SPSS 22. We corrected for multiple comparisons by controlling the false discovery rate using the Benjamini-Hochberg procedure (Benjamini and Hochberg 1995). To assess whether there was a stronger influence of age in the HIV group, these analyses were then re-run with the addition of an HIV × age interaction term.

To define the prevalence of cognitive impairment in the HIV and control groups, the domain *z*-scores were adjusted for age, years of education, and non-English speaking background. Individuals were then classified as cognitively “impaired” or “not impaired” using multivariate normative comparison (MNC) (Underwood et al. 2017; Castelli et al. 2010; Huizenga et al. 2007; Su et al. 2015) and Gisslen criteria (Gisslen et al. 2011). The MNC uses the cognitive profile for each member of the target (HIV) group in comparison with the distribution of scores in healthy controls, while accounting for covariance between tests: we employed the 5th percentile of the normal distribution as our criterion of abnormality, corresponding to a one-sided alpha value of 0.05 (compare Huizenga et al. (2007)). On the Gisslen criteria, participants were classified as impaired if they had a mean *z*-score < − 1.5 on two or more cognitive domains. The Gisslen criteria give a more conservative estimate, and we felt that this criterion was appropriate given the relatively small size of our samples.

To investigate whether cognitive performance was related to the putative explanatory factors, we ran a series of regression models that explored the influence of clinical, psychiatric, lifestyle, and neuroimaging variables on the four cognitive domains. All models adjusted for age, years in education, and non-English speaking background status. The following variables were explored: current CD4, nadir CD4, illness duration, treatment duration, time without treatment, CNS penetration effectiveness score, any metabolic comorbidity, BDI-II, BAI, weekly alcohol units, recreational drug use in the past three months (scored yes/no), and DTI metrics (MD and FA extracted from the whole brain white matter skeleton). In each model, we adjusted for the inclusion of multiple variables of interest using the Benjamini-Hochberg procedure (Benjamini and Hochberg 1995).

## Results

### Patient characteristics

Table 1 shows the background characteristics of the study participants. The control group differed from the HIV-positive group on the following variables: years of education, full-scale IQ, BDI-II, BAI, and PRMQ prospective memory score; although depression (BDI-II) and anxiety (BAI) scores were well within the “normal” range. In all our analyses, we covaried for years of education (which, in turn, are associated with full-scale IQ). The groups did not differ significantly on the other variables: age, non-English speaking background, PRMQ retrospective memory score, alcohol units per week, and drug use in the past 3 months.

### Group difference analysis for neuropsychological assessment

Table 2 shows the mean *z*-scores for cognitive performance on the various domains in the HIV-positive and control groups. Domain scores were adjusted for covariates (age, years in education, non-English speaking background).

**Table 2:** Mean (SD) *z*-scores for cognitive performance

	Control (*n* = 48)	HIV (*n* = 78)	Group difference effect size (Cohen’s *D*	Group difference *p* value	HIV × age group *p* value
Global cognition	0.00 (0.49)	− 0.46 (0.69)	0.53	*0.003*	0.856
Executive function	− 0.01 (0.60)	− 0.44 (0.81)	0.38	*0.030*	0.379
Complex attention	0.00 (0.61)	− 0.47 (0.88)	0.37	*0.040*	0.901
Memory	0.07 (0.63)	− 0.42 (0.78)	0.45	*0.014*	0.858
Perceptuo-motor function	0.00 (0.79)	− 0.68 (1.13)	0.46	*0.002*	0.445

All analyses controlled for age, years in education, and non-English speaking background

There were significant group differences on all domains (executive function, complex attention, memory, and perceptuo-motor function). These remained significant after correcting for multiple comparisons using false discovery rate (*p* < 0.05). There was no significant interaction between HIV status and age, indicating that the effect of age on cognitive performance did not differ between the groups in this cross-sectional study.

As significant differences in depression and anxiety scores were seen between the HIV-positive and HIV-negative participants, and these can have a marked influence on cognitive performance, the preceding analysis was repeated with BDI and BAI scores included as covariates. Effect sizes for the group differences were reduced in all domains and differences were no longer statistically significant for executive function (*d* = 0.27; *p* = 0.12) or complex attention (*d* = 0.27; *p* = 0.12) but remained significant for memory (*d* = 0.38; *p* = 0.03) and perceptuo-motor function (*d* = 0.41; *p* = 0.03). The differences are set in italics in Table 2.

### Prevalence of cognitive impairment

Individuals were classified as cognitively impaired or not impaired using MNC (Castelli et al. 2010; Huizenga et al. 2007) and Gisslen criteria (Gisslen et al. 2011). The MNC criteria classified 28% of the HIV impaired versus 5% of controls. On the Gisslen criteria, the equivalent figures were 13% versus 2%.

### Neuroimaging findings

**Fig. 1:**
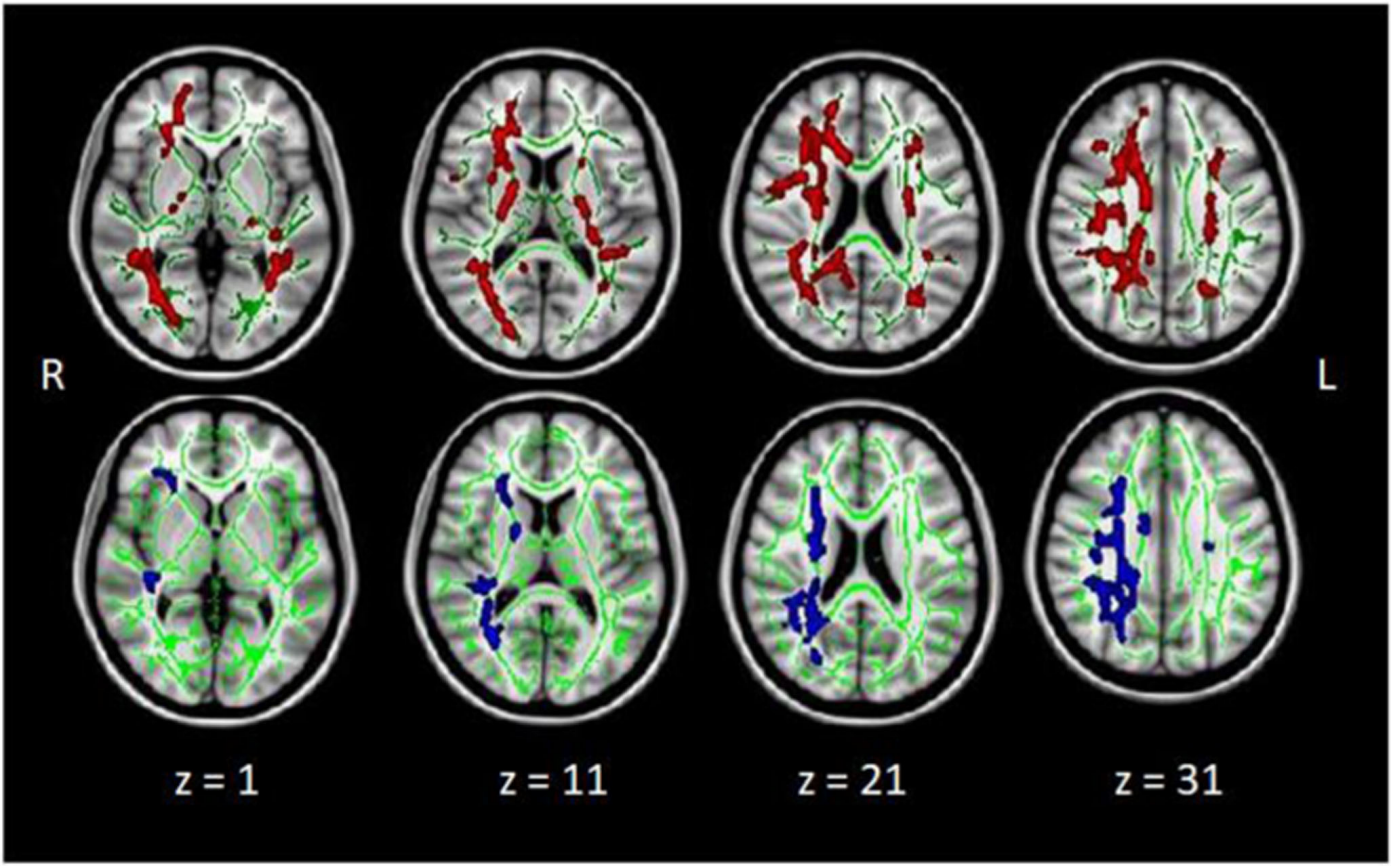
The influence of HIV status on fractional anisotropy (FA: top panel) and mean diffusivity (MD: bottom panel). Regions in which the HIV group showed lower FA (red) and higher MD (blue) than the seronegative controls (*p* < 0.05 threshold-free cluster enhancement corrected). This included the corpus callosum, corona radiata, internal capsule, and posterior thalamic in the right hemisphere for MD and bilaterally for FA

In the present sample, results of volumetric analyses did not survive cluster level correction for multiple comparisons. In contrast, on DTI metrics, the HIV group showed significantly higher MD and lower FA than the control group in the right corpus callosum, corona radiata, internal capsule, and posterior thalamic radiation (see Fig. 1). Group differences in FA were also seen in these regions in the left hemisphere.

### Analysis of effect of clinical, psychiatric, lifestyle, and imaging variables on cognitive function

Table 3 shows the findings after running a series of regression models in the HIV group to explore the influence of clinical, psychiatric, lifestyle, and neuroimaging variables on the four cognitive domains. Despite generally low levels of anxiety, this was the only variable to show a significant relationship with cognitive function after correcting for multiple comparisons. Higher anxiety was associated with lower scores on complex attention (*β* = − 386, *p* < 0.001) and executive function (*β* = − 381, *p* < 0.001). Using a more lenient significance level of *p* < 0.01, anxiety was also associated with lower memory scores (*β* = − 305, *p* = 0.005). Moreover, higher MD correlated significantly with lower executive function scores (*β* = − 304, *p* = .007) and lower FA with lower scores on complex attention (*β* = − 319, *p* = 0.005). Lastly, we re-ran the regression models for complex attention and executive function with anxiety and imaging metrics (either MD or FA) included in a single model. Both anxiety and the imaging parameters remained significant predictors of cognition, suggesting they were independently associated with cognitive performance (see Table 4).

**Table 3:** The relationship between clinical, psychiatric, lifestyle, and DTI variables and cognitive function

Variable	Memory		Complex attention		Executive function		Perceptuo-motor function	
	*β*	*p*	*β*	*p*	*β*	*p*	*β*	*p*
Current CD4	0.126	0.256	− 0.063	0.552	0.083	0.440	0.173	0.128
Nadir CD4	0.226	0.035	0.161	0.119	0.092	0.378	0.058	0.601
Illness duration	− 0.229	0.053	− 0.023	0.842	0.086	0.452	− 0.048	0.965
Treatment duration	− 0.124	0.319	− 0.062	0.600	0.059	0.601	− 0.142	0.266
Time without treatment	− 0.193	0.704	0.030	0.773	0.060	0.654	0.073	0.512
CPE score	− 0.216	0.048	− 0.057	0.558	− 0.047	0.659	− 0.014	0.900
Metabolic comorbidities	− 0.136	0.236	− 0.031	0.771	0.059	0.639	0.024	0.836
BDI	− 0.027	0.814	− 0.156	0.144	− 0.200	0.061	0.005	0.967
BAI	− 0.305	0.005	− 0.386	< 0.001	− 0.381	< 0.001	− .197	0.078
Weekly alcohol units	0.015	0.891	− 0.090	0.397	− 0.072	0.499	− 0.104	0.361
Drug use in past 3 months	− 0.040	0.717	− 0.038	0.716	− 0.048	0.652	0.003	0.979
Whole brain MD	0.040	0.696	− 0.257	0.026	− 0.304	0.007	− 0.213	0.093
Whole brain FA	0.075	0.542	0.319	0.005	0.247	0.029	0.259	0.040

All analyses controlled for age, years in education, and non-English speaking background

*CPE*, CNS penetration efficiency; *BAI*, Beck Anxiety Inventory; *BDI*, Beck Depression Inventory; *MD*, mean diffusivity; *FA*, fractional anisotropy

**Table 4:** The relationship between anxiety and DTI variables and cognitive function

	Complex attention		Executive function	
Variable	*β*	*p*	*β*	*p*
BAI	− 0.362	< 0.001	−0.315	0.001
Whole brain MD	− 0.263	0.012	−0.309	0.003
BAI	− 0.344	< 0.001	−0.297	0.003
Whole brain FA	0.297	0.005	0.228	0.032

All analyses controlled for age, years in education, and non-English speaking background

## Discussion

This cross-sectional study investigated whether HIV-positive participants, stable on cART, showed cognitive impairments, relative to HIV-negative controls. When the cognitive domains were analyzed separately, there were significant differences between the HIV-positive group and the seronegative control group across each cognitive domain (executive function, complex attention, memory, perceptuo-motor function, global cognition) after adjusting for age, years of education, and non-English speaking background. The difference was most marked for perceptuo-motor function although this did not correlate with any of the clinical or neuroimaging variables. When we examined the prevalence of cognitive impairment, we found a significantly higher rate in HIV-positive than HIV-negative participants when measured by either the MNC or Gisslen criteria. Our findings on the MNC criteria were similar to those obtained by Underwood et al. (2017): HIV-positive participants = 28% (Underwood et al. 19.5%) and HIV-negative participants = 5% (Underwood et al. 2.5%). Su et al. obtained rates of 17% and 5% by using MNC and Gisslen, respectively (Su et al. 2015). While Gisslen has better specificity and gives a more conservative estimate, MNC is thought to provide the optimal balance between sensitivity and specificity (Su et al. 2015).

Secondly, we explored the effect of clinical, psychiatric, and lifestyle variables on neurocognitive scores across these domains, covaried for age, years of education, and NESB. We found that clinically mild anxiety was the only factor that correlated significantly with cognition. There were significant correlations between this mild form of anxiety and both complex attention and executive function. The effect sizes for the differences in cognitive performance between the HIV-positive and HIV-negative participants were reduced when controlling for anxiety and depression, particularly in the complex attention and executive function domains (Table 2). This highlights the influence of anxiety on cognitive performance in this HIV group. It has previously been found that there is a high prevalence of clinical anxiety in people living with HIV (Janssen et al. 2015; Brandt et al. 2017), and that anxiety is associated with poorer performance on cognitive tests (Laverick et al. 2017), similar to findings of increased anxiety in mild cognitive impairment (MCI) (Chen et al. 2018). It has also been noted that there may be white matter abnormalities on DTI in people with anxiety-related disorders in the absence of HIV (Lochner et al. 2012; Lu et al. 2018). Taken together, these findings suggest that there may be a role for screening and treating anxiety and depression in patients with HAND (Seedat 2012; Spies et al. 2013; Wu and Li 2013; Sacktor et al. 2018).

We did not find any significant correlations between cognitive measures and metabolic factors, antiretroviral CPE score, or HIV infection severity. In part, this may have been because we excluded patients with unstable metabolic or HIV factors, but the finding is broadly consistent with that of Cole et al. (2017). A prospective study by Cross et al. (2013) also found no association between the antiretroviral CPE score and cognitive outcome, and they also excluded patients with uncontrolled medical conditions.

On DTI, the HIV-positive group showed higher MD and lower FA than the control group. This is in keeping with some other studies (Underwood et al. 2017; Cole et al. 2018; Su et al. 2015; O’Connor et al. 2017) and suggests that HIV infection is associated with greater changes in cerebral white matter and functional connectivity (Egbert et al. 2018), even in patients who are virologically suppressed on cART. Myelin damage has been found in central white matter in the pre-antiretroviral therapy era (Gray et al. 1992). Like Li et al. (2018), we found that the HIV group had increased MD in the corpus callosum and corona radiata among other regions. It has been suggested that these regions, being periventricular, are more vulnerable to viral invasion and neuro-inflammation in early infection when HIV infiltrates the CSF and by extension the white matter tracts (Li et al. 2018; Ragin et al. 2015). A meta-analysis by O’Connor et al. (2017), while only revealing a small reduction in FA in HIV, with high study heterogeneity, revealed a significant increase in MD in HIV.

It is possible that the found diffusion abnormalities in the HIV patients may have emerged from the myelin damage before the initiation of antiretroviral therapy (Underwood et al. 2017) or may reflect persisting immune activation and neuro-inflammation (Anzinger et al. 2014). Consistent with this latter hypothesis, our previous 4-year longitudinal investigation demonstrated significant correlations, on regression analysis, between change in global cognition, and changes in DTI measures during that period (Haynes et al. 2018).

In the present investigation, we compared age groups in a one-off cross-sectional comparison and did not obtain an HIV by age group interaction effect on cognition. In our previous investigation, Haynes et al. (2018) looked longitudinally at the cognitive decline over four years, obtaining an age-group by HIV interaction. These findings are not contradictory, resulting from the different methods employed, but they do suggest that if we conducted a cross-sectional study in much older participants, then we might indeed obtain a cross-sectional age group by HIV interaction effect on cognition, something we would like to examine in the future.

In summary, in this cross-sectional study, we found significant differences between the HIV group and their controls across four cognitive domains (executive function, complex attention, memory, perceptuo-motor function) after covarying for years of education. This was associated with abnormalities on DTI measures (FA, MD). We also found a correlation between subtle degrees of anxiety and three of our four cognitive domains, but not between other clinical or metabolic variables and cognitive function. Overall, our findings (as delineated in Table 4) suggested that both white matter changes (FA and MD on DTI) and subtle degrees of anxiety contributed independently to cognitive impairment in HIV.

Our study had several strengths. We did thorough neuropsychological testing and quantitative MRI assessments. The HIV-positive participants were all virologically suppressed on antiretroviral therapy. The groups were matched in terms of age, non-English speaking background, alcohol consumption, and drug use. On imaging, we found a difference between the HIV-positive and the seronegative groups. However, the study had a limitation: patients with unstable medical comorbidities were excluded, which may have led to an under-estimation of the effect of metabolic comorbidities on cognitive impairment. Studies which did not exclude these patients have found an association between neurocognitive impairment and metabolic factors, such as truncal obesity, diabetes, hyperglycemia, and insulin resistance, especially in older patients (Valcour et al. 2004; Valcour et al. 2006; McCutchan et al. 2012).

In conclusion, various studies have investigated white matter integrity in HIV and the effect of clinical variables on cognitive performance, but findings remain inconsistent. Our findings contribute to the debate, highlighting the importance of anxiety and DTI changes on cognitive function, and indicating the independence of their effects. We found a higher prevalence of neurocognitive impairment in our HIV group, and this was associated with higher MD and lower FA on neuroimaging. There was also a significant correlation between subtle levels of anxiety and cognitive impairment in the HIV group. Regular monitoring of neurocognitive functions and levels of anxiety should be conducted in HIV-positive people. Screening may help identify patients who would benefit from further psychological assessment and management of any anxiety.
